# Multi modal imaging in corneal edema after corneal collagen cross-linking (CXL); a case-based literature review

**DOI:** 10.1186/s12886-021-02220-x

**Published:** 2021-12-24

**Authors:** Mohammad Soleimani, Zohre Ebrahimi, Mohammad Yazdani Moghadam, Mansoor Shahriari, Sara Behzadfar, Bahareh Ramezani, Kasra Cheraqpour

**Affiliations:** 1 Eye Research Center, Farabi Eye Hospital, Tehran University of Medical Sciences, Tehran, 1336616351 Iran; 2 Imam Hossein Medical Center, Shahid Beheshti University of Medical Sciences, Tehran, Iran; 3 School of Medicine, Iran University of Medical Sciences, Tehran, Iran

**Keywords:** Corneal edema, Collagen cross-linking, CXL, Review, Keratoconus

## Abstract

**Background:**

Keratoconus (KCN) is a common ectatic disorder of the cornea. Corneal collagen cross-linking (CXL) is used as an effective option to slowdown the disease progression. Although CXL is considered a safe procedure, corneal endothelial damage, especially in corneal thickness of less than 400 μm, has been reported.

**Case presentation:**

A 25-year-old man known case of KCN was referred with complaints about blurred vision and discomfort of the right eye 3 days after performing CXL. The preoperative thinnest point was 461 μm. His presenting BCVA was CF at 1 m. Examination showed central corneal edema and stromal haziness. ASOCT demonstrated increased central corneal thickness and very deep CXL line. In the confocal scan, anterior stroma showed hyper-reflective lines without recognizable cells and nerves, the middle stroma showed rare active and edematous keratocytes and a hyper-reflective reticular pattern with elongated keratocytes and needle-like structures involving the posterior stroma indicated increased depth of CXL. To manage the patient, debridement of loosened epithelium was done. Non-preservative steroid 1% eye drop was prescribed frequently. The corneal edema was completely resolved during 2 months with no need for surgical procedure and BCVA of 20/30 in his right eye.

**Conclusion:**

The corneal thickness of more than 400 μm cannot guarantee the absence of corneal edema after corneal collagen cross-linking, which can pertain to several factors such as inadvertently using of higher energy as well as the incorrect observance of all guidelines, instructions, and other precautions, even by a trained surgeon.

## Background

Keratoconus (KCN) is a common ectatic disorder of the cornea with clinical pictures of progressive stromal thinning and highly irregular astigmatism leading to significant visual disturbance [1]. Nowadays, corneal collagen cross-linking (CXL) is used as an effective option to slow the disease progression. In this technique, riboflavin sensitivity to ultraviolet radiation strengthens the corneal biomechanic through providing additional cross-links between collagen fibers. These interactions can enhance the stiffness of corneal collagens and increase resistance against keratectasia [2, 3]. CXL is a highly safe procedure in the condition of adherence to standard protocols. However, several complications have been reported, which include persistent corneal haziness, infectious keratitis, and corneal endothelial cell damage, particularly at the corneal thickness of less than 400 μm [4].

Herein, we report a case of post CXL corneal edema in a patient who interestingly had preoperative corneal thickness of 461 μm. Also, we discuss findings of multimodal imaging of our case and, finally, provide a brief review on similar reported studies.

## Case presentation

**Fig. 1:**
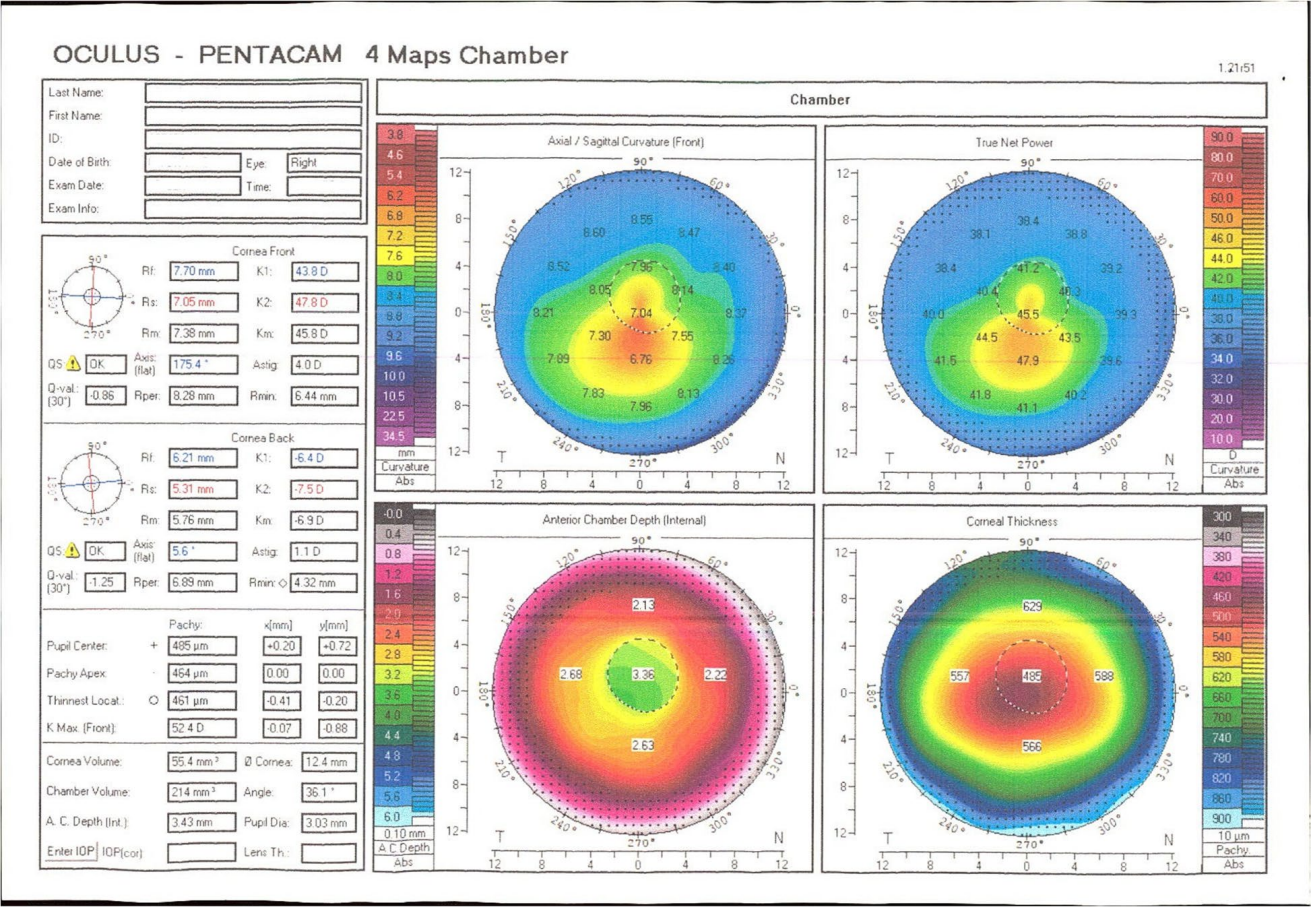
Preoperative Pentacam of the patient’s right eye

A 25-year-old man was referred with complaints about blurred vision and discomfort of the right eye 3 days after performing CXL in another ophthalmic center. He was a known case of KCN. Serial preoperative Scheimpflug tomography images indicated the progressive KCN. Also, the thinnest point of the right cornea was 461 μm, which allowed planning for the CXL procedure (Fig. 1). According to the patient’s medical records and through a phone call to his surgeon, preservative free 0.1% riboflavin solution with 20% dextran (Sina Darou, Iran) was administered every 3 min for 30 min after central 8-mm epithelial debridement. However, a crater or container was not used to keep the riboflavin in touch with the cornea. Then, this central part of the cornea was irradiated with UV-A light (IROC, Zürich, Switzerland) with the wavelength of 370 nm and radiation of 3.0 mW / cm^2^ after proper calibration. Riboflavin solutions were applied to the cornea every 5 min during the final 30 min of the procedure. Finally, a therapeutic contact lens was applied.

**Fig. 2:**
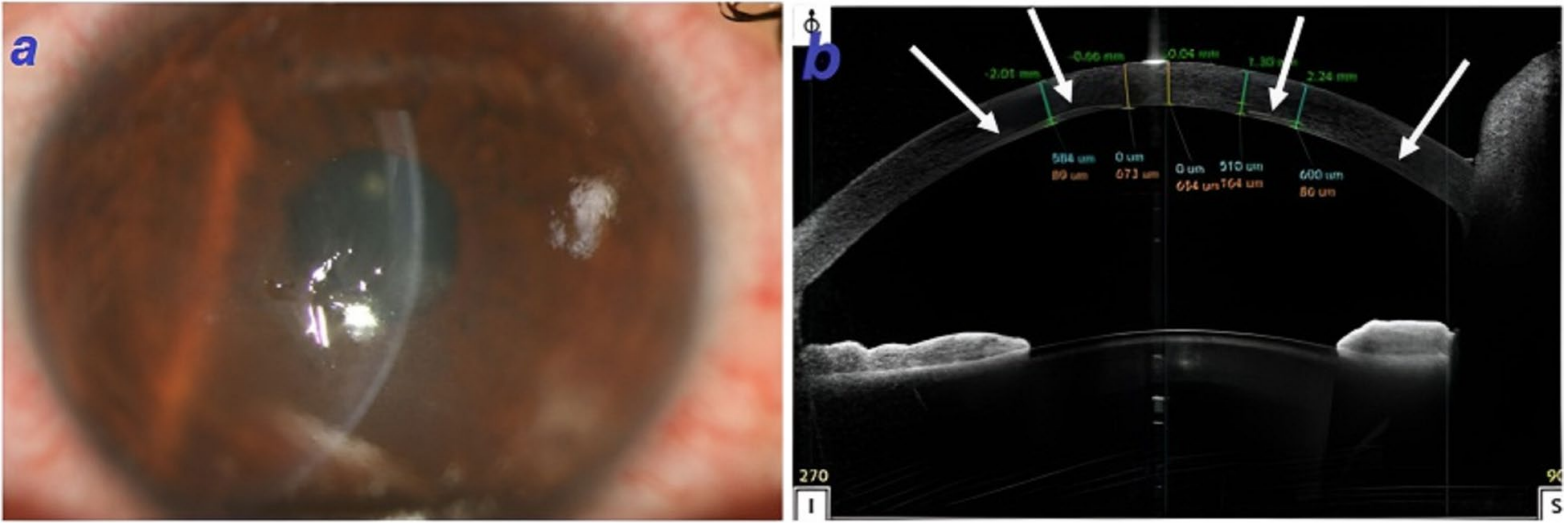
**a** Slit photograph of the right eye after CXL illustrates corneal edema and epithelial defect. **b** ASOCT of the right eye indicating increased thickness of cornea (white arrows are representative for faint demarcation line)

The patient developed corneal edema within the first day of procedure which did not resolve until the 3rd day of follow-up visit. Hence, the patient was referred to our center. He was suffering from pain and photophobia. Congestion, central corneal edema, and stromal haziness were noted on the slit-lamp examination (Fig. 2a). The therapeutic contact lens was in place. The crystalline lens was clear and the fundus examination was normal. The best-corrected visual acuity (BCVA) and manifest refraction of the right and the left eyes were counting fingers at 1 m (− 3.00–3.75 × 160 °) and 20/20 (− 2.00–0.25 × 47°), respectively. Furthermore, the intraocular pressure (IOP) of the right and the left eyes were 12 mmHg and 14 mmHg, respectively.

Different imaging modalities were applied. ASOCT (Optovue RTVue device, CA, USA) demonstrated increased central and paracentral corneal thickness (673 μm in the central part) with full-thickness stromal haziness. Moreover, no obvious Descemet rupture was detected. Demarcation line of CXL was visible as a faint hyper-reflective line between corneal stroma and endothelium in the paracentral area and beneath the endothelial layer in the center of the cornea showing a very deep CXL line (Fig. 2b).

**Fig. 3:**
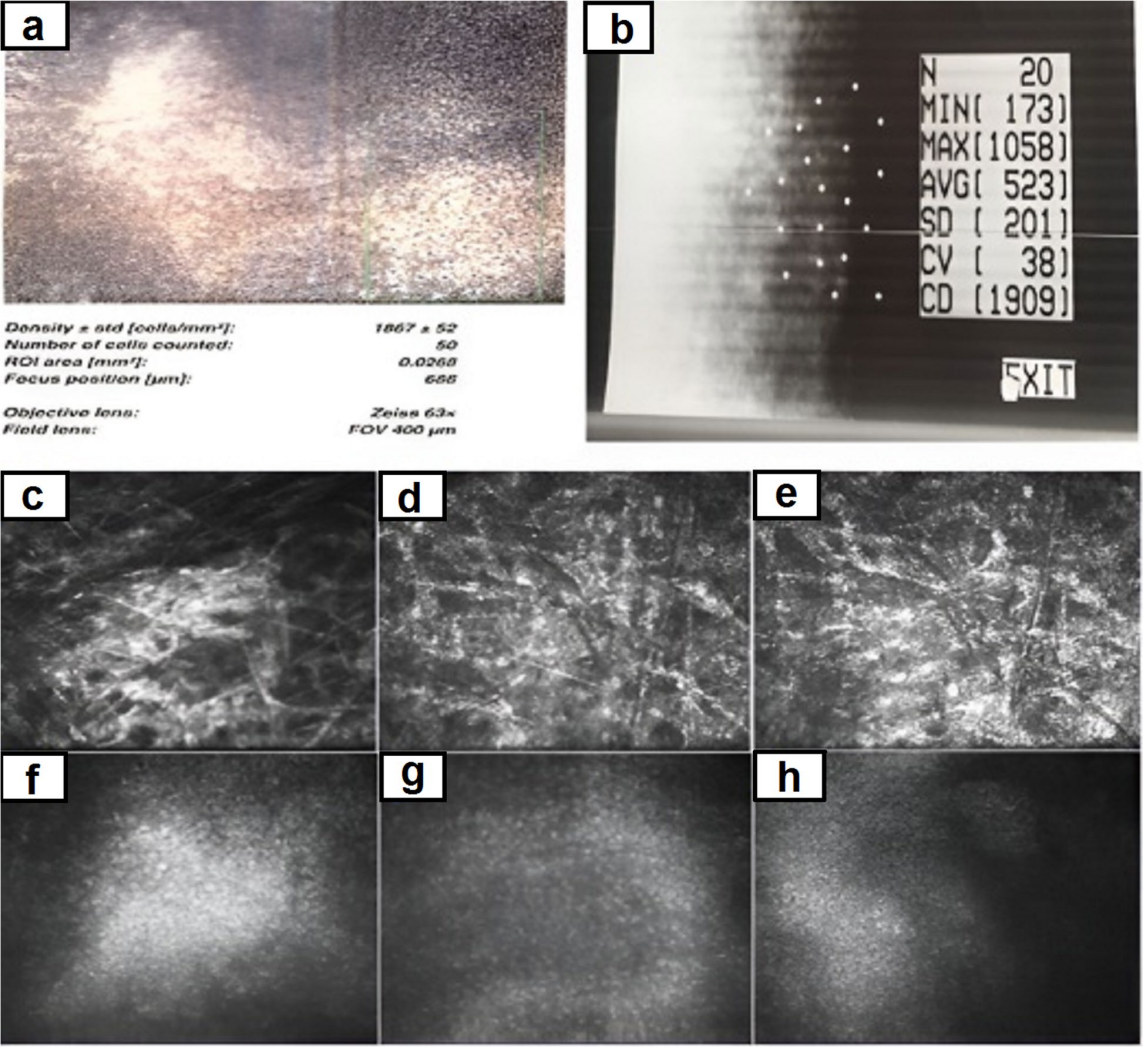
**a** Confocal scanning of the right eye estimated the number of corneal cells of about 1867 cells. **b** Specular microscopy of the right eye one month after treatment. **c** Confocal scan of the anterior stroma. **d** Confocal scan of the middle stroma. **e** Confocal scan of the posterior stroma. **f**,**g**,and **h**. Confocal scan of the endothelium

Although applying specular microscopy was not possible due to severe corneal edema, confocal scanning estimated the number of endothelial cells up to 1867 cells/m2 (Fig. 3a). It should be mentioned specular microscopy (SP 3000P; Topcon, Japan) done 1 month later when the cornea turned clear and revealed the cell density of 1909 cells /m2 (Table 1).

**Table 1:** Comparison of specular microscopy indexes between two eyes after resolving corneal edema

	Right eye (treated)	Left eye (untreated)
MIN	173	189
MAX	1058	378
AVG	523	315
CV	38	26
CD	1909	3207

*MIN* Minimum cell size, *MAX* Maximum cell size, *AVG* Average cell area, *CV* Coefficient of variation, *CD* Cell density

The confocal microscopy examination was performed for evaluating the effect of CXL on the different layers including anterior, middle, and posterior stroma, as well as endothelial layer. Anterior stroma showed hyper-reflective lines without recognizable cells and nerve fibers. In the middle stroma, rare active and edematous keratocytes were found. The boundary of the cross-sectional area was determined by a hyper-reflective reticular pattern with elongated keratocytes and needle-like structures that involved the posterior stroma, indicating the increased depth of the affected region of the CXL (Fig. 3).

**Fig. 4:**
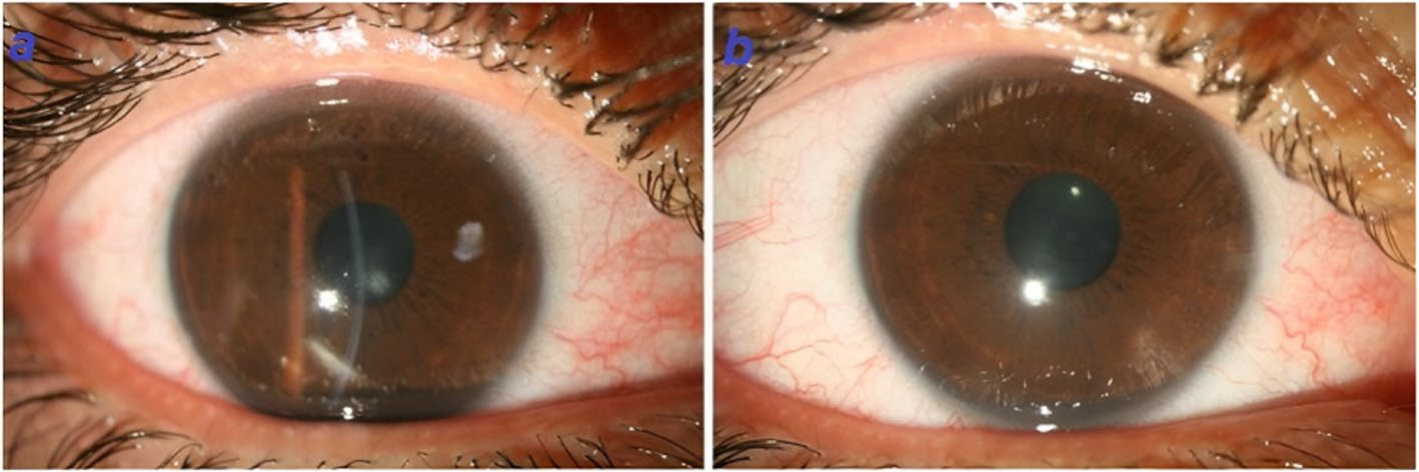
**a.** 4 days after treatment, epithelialization occurred and stromal edema reduced. **b.** Two months after treatment

To manage the patient, the bandage contact lens was removed followed by debridement of loosened epithelium for better re-epithelialization and substituted with new contact lens (FREQUENCY®55 soft contact lens with base curvature of 8.7 mm, diameter of 14.4 mm, and power of − 0.25 diopter). Non-preservative steroid 1% (NPS1%) eye drop was prescribed for every 2 h and homatropine 2% eye drop was used twice a day. On the 4th day of follow-up visit (7 days after CXL) re-epithelialization of the cornea was obviously accompanied by reduced edema and haziness (Fig. 4a). The corneal edema slowly reduced and completely resolved with no need for surgical procedures during 2 months. The stromal haze was partially resolved within 3 months with a residual whorl-shaped corneal scar (Fig. 4b). Steroids eye drops were tapered and discontinued in 3 months. Topographic evaluation was stable without any signs of progression. Finally, the patient obtained the BCVA of 20/30 in his right eye. It could be mentioned the preoperative refraction and BCVA were − 3.50 -4.00 × 160° and 20/25, respectively.

## Discussion and conclusions

Corneal collagen cross-linking is considered a favorable treatment option to slowdown the progression of keratoconus. This method is considered highly safe without significant damages to the endothelium or inner structures of the eye such as crystalline lens and retina in terms of adherence to standard protocols, indications, and contraindications. It has been shown that keratocyte loss within 300 μm of the stroma occurs immediately after the procedure, which takes about 6 months until repopulation [5].

The cytotoxic effect of riboflavin/UV-A combination on corneal endothelium has been revealed previously [6]. It seems human corneal endothelial cells are much more resistant to riboflavin-enhanced UV-A radiation than the animal corneal endothelial cells [7]. This finding may be related to the presence of collagen fibers which act as a protective factor. Moreover, thickness of 400 μm for saturation with riboflavin reduces the amount of radiation to the endothelial cells into level of 0.18 mW / cm^2^, which is half of the required level for cytotoxic effect (0.36 mW/cm^2^ (0.65 J/cm^2^) [5]. Hence, the corneal thickness of less than 400 μm has been identified as the most important risk factor for developing post CXL endothelial cell damage [6].

Several factors have been introduced that might play a role in endothelial damage including inadvertent delivery of excessive energy due to poor calibration and focusing, intraoperative corneal thinning secondary to dehydration caused by epithelium removal or application of dextran containing riboflavin drops, inaccurate pachymetry reading during surgery, acute hydrops, pre-existing Fuchs endothelial dystrophy, extreme intra-CXL treatment of corneal thinning occurring during CXL, and endothelial cell damage due to waterjet wave during intraoperative irrigation procedure [8]. Also, herpetic disciform edema and noninfectious endotheliitis caused by direct injury to the endothelial cells by UV-A are the other possibilities [9]. However, clinical and imaging findings of our case were not compatible with those of the two latter differential diagnoses. To the best of the author’s knowledge, only 4 reports (containing 13 cases) of post CXL corneal edema are available on the Pubmed/Medline database, as summarized in Table 2.

**Table 2:** Review on similar reported reports regarding post CXL corneal edema

Number [Ref.]	Age Sex Eye	Elapsed time after CXL to presentation	Preoperative VA, Keratometry, Pachymetry	Riboflavin usage	Examination	Investigation	Outcome
1 [8]	18 M OD	20 days	20/20 50 514	Riboflavin (0.1%) in dextran 20% every 2 min for 30 min which continued every 2 min during irradiation	-Epithelial and stromal edema with Descemet folds -Pigmented KPs -Dilated and fixed pupil	-Pachymetry: thickness more than 1000 mm -Negative PCR of aqueous fluid -ECD: 1571	-Performing of PK 6 months later due to epithelial bullae and severe corneal edema - BCVA of 20/60 three months after PK
2 [10]	37 M OS	1 month (start of symptoms was since the first postoperative day)	20/60 57.8 448	0.1% riboflavin every 5 min for 25 min which continued every 5 min during irradiation	-Central corneal edema with severe stromal haze -No Descemet rupture	-ASOCT: thickness of 844 mm -Specular microscopy: 1776 (at the 6-month visit)	-Resolved edema in 3 months -Resolved stromal haze in 6 months -A residual ring-shaped corneal scar -BCVA of CF at 3 m
3 [9]	24 F OD	3 days	20/40 53.4 488	0.1% riboflavin with 20% dextran every 2 min for 30 min which substituted with 0.1% hypotonic riboflavin every 2 min during irradiation to swell the cornea to at least 400 mm	-Ring-shaped endothelial infiltrates and localized corneal edema in the inferior paracentral of cornea	-ECD of both eyes were comparable after resolving of the problem (2732 vs. 2707)	-Resolved after 1 week -BCVA of 20/25 (lens-fitted)
4 [11]	Average of age: 22 ± 5 7 M and 3 F (10 patients)	First day (in all cases)	Median logMAR of BCVA: 0.00 ± 0.08 Median of maximum keratometry: 57.4 ± 2.90 Mean of pachymetry: 472.6 ± 17.5	Isotonic riboflavin 0.1% every 2 min for 30 min which continued every 3 min during irradiation	-Corneal edema and anterior chamber inflammation which increased for 2 to 3 weeks –No Descemet membrane rupture -No lens opacities -Corneal vascularization (2 eyes) -Iris atrophy (6 eyes) -Pigment dispersion (5 eyes) -CED for more than 6 days (3 eyes) -Infectious keratitis (1 eye)	N/A	-Median logMAR of BCVA: 0.60 ± 0.30 -Corneal edema improved in 4 patients and resolved in 1 patient. − 2 patients underwent PK at 8 and 13 months after CXL

*F* Female, *M* Male, *OD* Right eye, *OS* Left eye, *KP* Keratic precipitate, *PCR* Polymerase chain reaction, *ECD* Endothelial cell density; *PK* Penetrating keratoplasty, *BCVA* Best-corrected visual acuity, *ASOCT* Anterior segment optical coherency tomography, *CF* Counting fingers, *CED* Corneal epithelial defect)

Our case developed corneal edema immediately after the procedure which completely was resolved within further follow-up visits. Multimodal imaging such as confocal microscopy, specular microscopy, and ASOCT was performed to evaluate corneal edema. Confocal microscopy revealed the corneal endothelial cell density of about 1860 cells which was 60% of cell density of the untreated fellow eye (Table 1). Furthermore, specular microscopy after clearing the corneal edema showed decreased number of endothelial cells, polymorphism, and polymegatism considered as other witnesses of endothelial cell damage during this procedure. However, endothelial cells count before CXL was unknown, but based on medical records and the referring surgeon’s claim, preoperative clinical examination did not reveal any endothelial abnormalities.

The ASOCT showed an increase in the depth of cross-linking, in which the demarcation line was observed in the posterior part of the stroma and the center area on the endothelium. However, it normally should be detected between the anterior third and posterior two-third of the stroma. Other causes of corneal edema such as acute hydrops and rupture of the Descemet membrane were ruled out by this imaging.

In addition to decrease in the number of endothelial cells in the acute phase, confocal microscopy showed the effects of cross-linking in the form of hyper-reflective lines with a reticular pattern at posterior stroma and endothelium (depth of 456 μm), which was another confirmation of the increase in cross-linking depth. Also, it seems needle-like structures are representative for new synthesized collagen produced by activated keratocytes at the transition zone [12]. Although the referring surgeon insisted on following the standard protocols, an error in performing cross-linking can be considered as one of the possible causes for this complication. Furthermore, it seems one of the pitfalls in the management of this patient is lack of intraoperative pachymetry since it has been shown remarkable alteration in pachymetry is not an uncommon event during CXL.

Improper focus or calibration of the ultraviolet light source and closer position of the eye in machines using only one light-emitting diode (LED) as the UV-A source can cause delivery of higher energies and subsequent cytotoxic damages to the corneal endothelium. Moreover, the presence of intact film of riboflavin during the procedure is so important. On the one side, riboflavin acts as a photosensitizer increasing the absorption of UV, which results in collagen cross-linking. On the other side, riboflavin has a protective role blocking the delivery of high energy to inner structures like endothelium. Moreover, continuity in providing riboflavin prevents from dehydration and subsequent corneal thinning [10]. It has been reported dextran–riboflavin solution can lead to about 10% corneal thinning [13]. So, frequent instillation of this agent during the procedure should be considered.

Beside the corneal thickness, proper calibration and focusing, removal of the lid speculum during instillation of riboflavin drops to prevent excessive thinning secondary to evaporation, providing intact continuous film of riboflavin, transepithelial CXL, accelerated CXL, and frequent intratreatment checks of the corneal thickness with re-administration of hypotonic solution if the thickness drops to less than 350 μm are the considerable factors for preventing from collateral damages to other structures of the eye such as endothelium [14].

In conclusion, our case highlights that the corneal thickness of more than 400 μm cannot guarantee the absence of corneal edema after corneal collagen cross-linking, which can pertain to several factors such as inadvertently using higher energy due to improper calibration or focus as well as the incorrect observance of all guidelines, instructions, and other precautions, even by a trained surgeon.

## Data Availability

The data are available from the corresponding author on reasonable request.

## Abbreviations

KCN: Keratoconus
CXL: Corneal collagen cross-linking
CCT: Central corneal thickness
BCVA: Best-corrected visual acuity
UV: Ultra violet
NPS: Non-preservative steroid
LED: Light-emitting diode
